# Transforming Medical Library Staff for the Twenty-First Century

**DOI:** 10.5195/jmla.2018.510

**Published:** 2018-10-01

**Authors:** Elizabeth Connor

**Affiliations:** Daniel Library, The Citadel, Charleston, SC

The challenges of leveraging and developing health sciences library staff to support new and evolving institutional and professional directions are the focus of this handy and useful work. Librarians with current and past experience working in private and public settings in Arizona, Connecticut, Indiana, Iowa, Louisiana, Maryland, Massachusetts, South Carolina, Utah, and Virginia have contributed chapters and provided practical examples and insights of value to other librarians interested in organizational transformation. If you have ever wondered how to do more with the same resources or fewer, this book is an excellent starting point. Library staff members are our most valuable and renewable resources.

National Library of Medicine Director Patricia Flatley Brennan’s foreword is followed by ten chapters that emphasize understanding the changing environment for health sciences libraries (Yale University); empowering staff (Virginia Commonwealth University); developing skills (Indiana University-Purdue University Indianapolis, University of Maryland at Baltimore, National Network of Libraries of Medicine Greater Midwest Region); understanding the qualities of nimble organizations (University of Utah, University of Maryland at Baltimore); communicating effectively (Medical University of South Carolina); managing union employees (University of Massachusetts Medical School); knowing staff well enough to appreciate generational differences and identify strengths (Louisiana State University Health Shreveport); understanding staff readiness (University of Arizona); working in one-person libraries or solo librarians (Boston Children’s Hospital, Brigham & Women’s Hospital); and recruiting, retaining, and rewarding employees through appreciative inquiry (Medical University of South Carolina).

The work is timely and readable, and can be consulted by specific chapter or consumed cover to cover. The biographies of chapter contributors reads like a “Who’s Who” of the Medical Library Association, evidenced by their depth and breadth of experience. Each chapter is enhanced with cited references and additional suggested readings. A six-page index aids in finding specific topics.

This book is highly recommended for all health sciences librarians, but with the book’s emphasis on academic medical centers, hospital librarians might not find enough content here for their purposes.

